# Increased Visibility of Deep Medullary Veins in Leukoaraiosis: A 3-T MRI Study

**DOI:** 10.3389/fnagi.2014.00144

**Published:** 2014-06-30

**Authors:** Shenqiang Yan, Jinping Wan, Xuting Zhang, Lusha Tong, Song Zhao, Jianzhong Sun, Yuehan Lin, Chunhong Shen, Min Lou

**Affiliations:** ^1^Department of Neurology, The Second Affiliated Hospital of Zhejiang University, School of Medicine, Hangzhou, China; ^2^Department of Radiology, The Second Affiliated Hospital of Zhejiang University, School of Medicine, Hangzhou, China

**Keywords:** white matter hyperintensities, leukoaraiosis, susceptibility-weighted imaging, deep medullary veins, venous ischemia, aging

## Abstract

Cerebral venous collagenosis has been implicated in leading to white matter hyperintensities (WMHs) via venous ischemia. We sought to determine whether cerebral venous dilation or ischemia correlate with the severity of WMHs by quantitative *in vivo* imaging techniques. This was an investigator-initiated prospective single-center study. We reviewed clinical, laboratory data from 158 consecutive WMHs patients and 50 controls, and measured the number of voxels of deep medullary veins (DMVs) on susceptibility-weighted image and assessed the WMH volume (as a marker of the severity of WMHs) on a 3-T magnetic resonance system. We then performed the logistic-regression analysis and partial Pearson’s correlation analysis to examine the association between the venous voxel count and WMH volume. The number of voxels of DMVs was significantly higher in WMHs than in controls. Increased number of voxels of DMVs was independently associated with both WMH volume of the whole brain and coregistered regional WMH volume after adjusting for age and number of lacunes. Our study indicates that cerebral deep venous insufficiency or ischemia play a role in the pathogenesis of WMHs, which may provide prognostic information on patients with WMHs and may have implications for therapeutic interventions.

## Introduction

White matter hyperintensities (WMHs), also referred to as leukoaraiosis, are commonly seen as patchy or confluent hyperintense areas on T2 weighted or fluid attenuated inversion recovery (FLAIR) magnetic resonance imaging (MRI) scans in the elderly population. Their presence is associated with an increased risk of stroke, dementia, and mortality (Debette and Markus, 2010). Deficits of information processing speed and executive function were also reported to be correlated with the severity of WMHs (O’Sullivan et al., 2004; Prins et al., 2005). Despite its importance, the pathogenic mechanisms underlying WMHs is so far unclear.

The development of WMHs has been related to the diminished regional cerebral blood flow (CBF), disruption of capillary permeability, and a damaged blood-brain barrier (BBB) (Akiguchi et al., 1998; Brickman et al., 2009). Recently, emerging literatures suggest that cerebral venous collagenosis may cause venous ischemia by increasing vascular resistance and compromising interstitial fluid circulation, with consequent vessel leakage, i.e., vasogenic edema, and lead to non-necrotic hyperintensities on MRI (Moody et al., 1995; Black et al., 2009). The small veins within the white matter of the cerebral hemispheres, referred to as “deep medullary veins (DMVs),” directly participate the drainage of white matter via the subependymal veins into the internal cerebral vein or the basal vein of Rosenthal (Huang and Wolf, 1964). Cerebral venous hypertension and venous outflow insufficiency may lead to dilation of DMVs and play a role in the pathogenesis of WMHs. However, only a few postmortem studies have addressed it to date, and there are no quantitative *in vivo* imaging techniques aimed to characterize their possible relationship.

Susceptibility-weighted imaging (SWI) (Haacke et al., 2004), through the unique use of both magnitude and phase images from a high-resolution, three-dimensional fully velocity compensated gradient recalled echo (GRE) sequence, is an improved MRI technique that can depict small vessels and venous structures rich in deoxygenated blood. Using deoxyhemoglobin as an intrinsic contrast agent, SWI venography affords a non-invasive assessment of cerebral veins. Therefore, in the present study, we used SWI imaging to compare DMVs in patients with WMHs and control subjects, and then investigated the relationship between voxel count of DMVs and the volume of WMHs. The main objective of this study is to explore whether increased voxel count of DMVs are associated with severity of WMHs. These quantitative measures of venous structures may be the key to understand the role of venous ischemia in the pathogenesis of the WMHs and allow the clinician not only to monitor the severity and progression of WMHs but also to evaluate the response to the therapies in WMHs.

## Materials and Methods

### Study subjects

This was an investigator-initiated prospective single-center study. We routinely reviewed the records of all patients admitted to our department, who received brain MRI scan but had no diagnostic intracerebral lesions such as acute stroke, trauma, infection, and space-occupying lesions from January 2010 to May 2013. We reviewed their brain MRI and identified WMHs as following: hyperintensities of caps around the anterior and posterior horns of the lateral ventricles, pencil-thin lining or a smooth halo along the side of the lateral ventricles, and punctate or beginning confluent or confluent changes in the subcortical areas. We then enrolled those who met all of the following inclusion and none of the exclusion criteria into this study.

Inclusion criteria were (i) WMH on MRI; (ii) age above 30; (iii) agreement to give written informed consent. Exclusion criteria were (i) patients with secondary causes of white matter lesions, such as immunological, demyelinating, metabolic, toxic, infectious, and other causes; (ii) patients with abnormal brain MRI findings such as space-occupying lesions, head trauma, hemorrhage, or infarction (except lacunes); (iii) patients with definitive peripheral neuropathy, spinal cord disease; (iv) abnormal hypointense lesions along the DMVs on phase images, such as microbleeds or hemorrhage; (v) evidence of calcification on the CT scans or encephalomalacia in the deep gray matter structures which may influence the calculation of DMVs.

We retrieved baseline demographic, clinical, laboratory, and radiological data including age, gender, years of education, the comorbid conditions such as history of hypertension, diabetes mellitus and hyperlipidemia, systolic blood pressure (SBP), and diastolic blood pressure (DBP), serum glucose level, total cholesterol, total homocysteine and high-sensitivity C-reactive protein, and number of microbleeds and lacunes on MRI. All patients underwent a mini-mental state examination (MMSE) (Folstein et al., 1975).

Fifty healthy adults served as controls for the visibility of DMVs after giving written informed consent. They were recruited for our previous study (Yan et al., 2012) or served as volunteers for our ongoing fMRI projects. Their clinical data, laboratory examinations (for common vascular risk factors such as hypertension, diabetes mellitus and hyperlipidemia), and radiological examinations (both CT and MRI) were normal. The age and gender were matched with the WMHs group.

### Ethics statement

All subjects had given written informed consent prior to the study, and the protocols had been approved by the local ethics committee. All clinical investigation has been conducted according to the principles expressed in the Declaration of Helsinki.

### MRI parameters

All subjects underwent multi-modal MRI including T1, T2, T2 FLAIR, and SWI sequence on a 3.0 T system (Signa Excite HD, General Electric Medical System, Milwaukee, USA) equipped with an eight-channel phased array head coil. Foam pads were inserted into the space between the subject’s head and the MRI head coil to minimize head motion. Axial T2 FLAIR sequence was used to measure the WMH volume with the following parameters: repetition time = 10000 ms, echo time = 150 ms, FOV = 24 cm × 24 cm, matrix size = 256 × 256, inversion time = 2500 ms, slice thickness = 5.0 mm with no gap (continuous) between slices, and in-plane spatial resolution of 0.4688 mm/pixel × 0.4688 mm/pixel. The whole brain was imaged.

The SWI sequence was in an axial orientation parallel to the anterior commissure to posterior commissure (AC-PC) line and covered the whole lateral ventricles, using a three-dimension multi-echo gradient-echo sequence with 11 equally spaced echoes: echo time = 4.5 ms [first echo], inter-echo spacing = 4.5 ms, repetition time = 58 ms, FOV = 24 × 24 cm^2^, matrix size = 256 × 256, flip angle = 20°, slice thickness = 2.0 mm with no gap between slices, and in-plane spatial resolution of 0.4688 mm/pixel × 0.4688 mm/pixel. Flow compensation was applied. Magnitude and phase images were acquired, and all of the data were used in further analysis.

### Volumetric assessments of WMHs

We processed axial T2 FLAIR images for the quantification of WMH volume according to the published methods (Decarli et al., 1992, 1995). The segmentation threshold for WMHs was determined *a priori* as three standard deviations in pixel intensity above the mean of the fitted distribution of brain parenchyma as described previously (Decarli et al., 1995; Wen and Sachdev, 2004). Since the intracranial volume (ICV) of each individual was different, we measured the corrected WMH volume (CWMHV) for further analysis with the following equation: CWMHV = (WMHV × mean ICV)/ICV. The mean ICV is the mean value of ICV of all patients.

**Figure 1:**
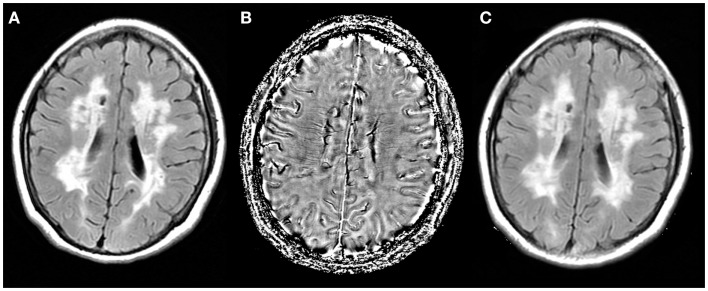
**Axial T2 fluid attenuated inversion recovery (FLAIR) images were coregistered to the phase images**. **(A)** T2 FLAIR image; **(B)** phase image of the same patient was set as a reference; **(C)** coregistered T2 FLAIR image.

We measured the volume of periventricular hyperintensity (PVH) (frontal horn, occipital horn, and lateral bands) and deep white matter hyperintensity (DWMH) (frontal, parietal, and occipital), respectively, considering that PVH and DWMH may differ with respect to pathophysiology (Fazekas et al., 1993; O’Sullivan et al., 2002). PVH volume was calculated 10 mm away from everywhere around the lateral ventricles for confluent type (Young et al., 2008). We also measured regional WMH volume, which was illustrated in Figure 1. Briefly, the axial T2 FLAIR images were coregistered to the phase images, and we calculated the WMH volume around the DMVs on the same slices used in the DMVs measurement (10 mm thick), corrected by the ICV, too.

### Number of voxels of the DMVs measurement

**Figure 2:**
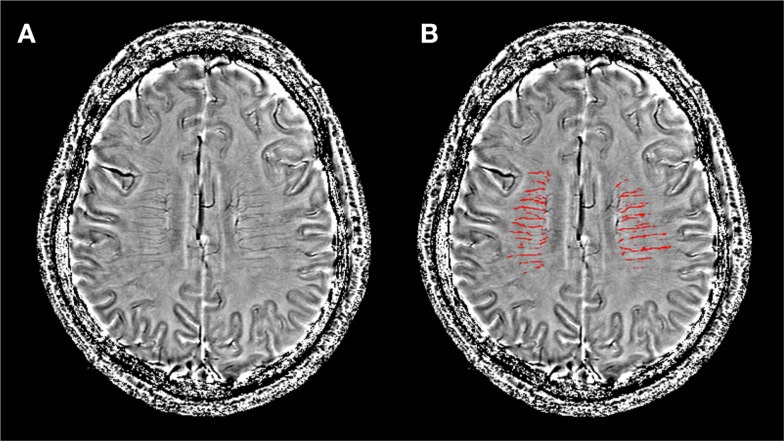
**Image post-processing and segmentation of the deep medullary venous structures were based on filtered phase images**. **(A)** A filtered phase slice of one WMH patient; **(B)** the same slice after segmentation used to calculate the number of voxels of the deep medullary veins for quantification.

The raw data were transferred to a separate workstation (ADW4.4, GE), where the magnitude and phase images were obtained by a custom built program. We used a high-pass filter with a central matrix size of 32 × 32 to remove background field inhomogeneities to create the corrected phase images, and then use MRIcro software (Chris Rorden, University of Nottingham, Great Britain) to detect the deep medullary venous vasculatures (Figure 2). We quantified the venous blood voxel count by segmentation of the vein and surrounding tissue on SWI phase data. The segmentation threshold for DMVs was determined as two standard deviations in voxel intensity below the mean of the fitted distribution of brain parenchyma. We measured five consecutive periventricular slices (10 mm thick) from the level of the ventricles immediately above the basal ganglia to the level of the ventricles immediately disappeared for each patient, considering that these slices cover most of the DMVs. The DMVs were distinguished from other venous structures and abnormal hypointense lesions, as described in detail elsewhere (Friedman, 1997). The false DMVs areas were visually identified by an experienced observer (Min Lou) and then manually removed with MRIcro. Finally, the number of voxels from bilateral veins was computed for quantification analysis. Minimum intensity projection (mIP) images were not taken in this study due to the absence of three-dimensional information of the veins.

### The presence of microbleeds and lacunes

We used SWI magnitude images to detect the presence of microbleeds in both hemispheres. Microbleeds were defined as small, rounded or circular, well-defined hypointense lesions within brain parenchyma with clear margins ranging from 2 to 10 mm in size on GRE images (Greenberg et al., 2009). Signal voids caused by sulcal vessels, calcifications, choroid plexus, and low-signal averaging from adjacent bone were excluded (Vernooij et al., 2008).

We used T2 FLAIR images to detect the presence of lacunes in both hemispheres. Lacunes were defined as cavities with a diameter of 3–10 mm with signal intensities similar to cerebrospinal fluid on MRI which was different from the enlarged Virchow–Robin spaces by the size, shape, and rim (Gouw et al., 2008).

### Reliability and validity of the radiological measurements

All the raters were blinded to all other imaging and clinical data. A single-trained observer (Shenqiang Yan) performed the quantitative assessments of 50 patients twice, at an interval of 3 months apart. Another observer (Jianzhong Sun) independently made the measurements on the same patients. The interobserver intraclass correlation coefficients (ICCs) were 0.97 for WMH volume and 0.93 for number of voxels of DMVs. The intraobserver ICCs were 0.95 for WMH volume and 0.90 for number of voxels of DMVs. ICCs were described in detail elsewhere (Lee et al., 1989).

### Statistical analysis

The patients and controls were age- and gender-matched, and patients were dichotomized according to WMH volume at the median. Fisher’s exact test was used to compare the dichotomous variables between groups, while independent samples two-tailed *t*-test was used for the continuous variables. We performed partial Pearson’s correlation analysis (that measures the degree of association between two random variables, with the effect of a set of controlling random variables removed) to determine the correlation between CWMHV, corrected PVH volume, corrected DWMH volume, and number of voxels of DMVs, by adjusting for age, sex, years of education, and number of microbleeds and lacunes (Baba et al., 2004). Venous occlusion has been demonstrated to lead to partially reversible WMHs in patients with arteriovenous malformations (van den Berg et al., 2008), we thus hypothesized that venous ischemia was more likely to be a cause of WMHs. In addition to the partial Pearson’s correlation, we also conducted logistic regression (small vs. large CWMHV) to provide an odds ratio statistic, thus facilitating comparison with other known risk factors. Backward stepwise conditional method was used to avoid the unstability resulted from collinearity of the included variables. Age was forced into the model, while other demographic variables (sex and years of education), number of voxels of DMVs, and variables with a *P* < 0.1 in univariate regression analyses were included in the backward stepwise conditional model. MMSE was not included as a consequence of the severity of WMH. Since the WMH volume was skewed toward the left of mean, we performed natural log transformations before the correlation analysis. The log-transformed WMH volume appeared to be acceptably normative. Bonferroni correction was used for comparison between multiple groups. All analyses were performed blinded to participant identifying information. Statistical significance was set at a probability value of <0.05. All statistical analysis was performed with SPSS package (14.0 for Windows).

## Results

### Subject characteristics

Totally, 158 consecutive WMH patients were enrolled in this study. The reasons for admission of those patients were mild cognitive impairment (*n* = 17, 10.8%), gait disturbances (*n* = 9, 5.7%), dizziness (*n* = 42, 26.6%), mixed reasons above [*n* = 28, 17.7%, four patients with additional urinary incontinence, reported in WMH patients (Poggesi et al., 2008)], other symptoms such as headache (*n* = 8, 5.1%) and transient ischemic attack which were not recurrent during the 3-month follow-up (*n* = 7, 4.4%), and incidental finding of WMHs on MRI without symptoms (*n* = 47, 29.7%). The age of the patients ranged from 30 to 88 years (mean = 66.51, SD = 12.76), and the CWMHV of the patients ranged from 2.58 to 134.63 ml (mean = 35.32, SD = 28.25). Among them, we found 76 patients with lacunes and 84 with microbleeds. The age of the 50 controls ranged from 40 to 78 years (mean = 66.21, SD = 12.13).

### Comparison of the DMVs between WMHs and controls

There were no significant differences in age and gender between WMHs and controls. The voxel number of DMVs in WMHs was significantly higher than controls (3675.79 ± 1071.29 vs. 2614.33 ± 1066.44, *P* < 0.001). There were no significant correlations between age and voxel count of DMVs in both patients (Pearson *r* = 0.049, *P* = 0.537) and controls (Pearson *r* = 0.124, *P* = 0.431).

### Univariate and multivariate regression analysis of the WMH volume

**Figure 3:**
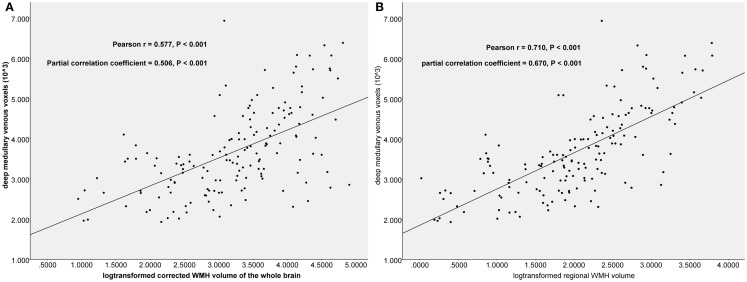
**The scatter plots between log-transformed corrected white matter hyperintensity (WMH) volume and number of the deep medullary venous voxels**. Partial Pearson’s correlation analysis was used by adjusting for age, sex, years of education, and number of microbleeds and lacunes. **(A)** The correlation between voxel count and WMH volume of the whole brain. **(B)** The correlation between voxel count and the regional WMH volume.

The CWMHV was significantly correlated with the voxel number of DMVs (Pearson *r* = 0.577, *P* < 0.001; partial correlation coefficient = 0.506, *P* < 0.001; Figure 3A), and this correlation was also significant when measured in left (Pearson *r* = 0.512, *P* < 0.001; partial correlation coefficient = 0.463, *P* < 0.001) and right hemisphere (Pearson *r* = 0.527, *P* < 0.001; partial correlation coefficient = 0.494, *P* < 0.001) after adjusting for age, sex, years of education, and number of microbleeds and lacunes, respectively. We also found a significant correlation between CWMHV and the number of lacunes (Pearson *r* = 0.327, *P* = 0.004; partial correlation coefficient = 0.290, *P* < 0.001) and microbleeds (Pearson *r* = 0.305, *P* = 0.005; partial correlation coefficient = 0.189, *P* = 0.019).

**Figure 4:**
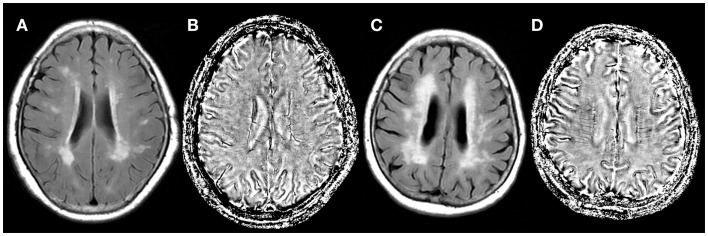
**Fluid attenuated inversion recovery images (A,C) and SWI phase images (B,D) of two (white matter hyperintensity) WMH patients**. The corrected white matter hyperintensity volume was 82.66 ml **(C)** and 16.88 ml **(A)**. Number of voxels of the deep medullary veins was increased (4897 vs. 2782) in the severe WMH patient **(D)**, compared with mild WMH patient **(B)**.

Table 1 shows the baseline characteristics of patients for comparison. Patients with large CWMHV had lower MMSE score and more number of microbleeds and lacunes, compared with those with small CWMHV. The voxel number of DMVs was higher in patients with large CWMHV (*P* < 0.001). Examples were given in Figure 4. Age, sex, years of education, number of voxels of DMVs, SBP, and number of microbleeds and lacunes were included in the primary logistic-regression model, and sex, years of education, SBP, and number of microbleeds and lacunes were then removed from the backward stepwise conditional model. Table 2 lists the results of backward stepwise conditional logistic-regression analysis. The voxel number of DMVs was an independent influencing factor for large CWMHV (>25 ml) (odds ratio = 3.229; 95% CI: 1.986 to 5.251; *P* < 0.001), after adjusting for age and number of lacunes.

**Table 1:** **Baseline characteristics of WMH patients classified according to corrected WMH volume dichotomized at the median**.

Variable	Corrected WMH volume		*P* value
	Small (≤25 ml, *n* = 79)	Large (>25 ml, *n* = 79)	
Age (y)	65.48 ± 13.32	67.54 ± 12.17	0.311
Female	39 (49.4%)	39 (49.4%)	1.000
Years of education (y)	6.76 ± 4.70	7.76 ± 4.44	0.171
MMSE	27.35 ± 1.79	24.30 ± 4.40	<0.001**
**PAST MEDICAL HISTORY**			
Hypertension			0.768
No	17 (21.5%)	21 (26.6%)	
Yes, with medication	46 (58.2%)	42 (53.2%)	
Yes, without medication	16 (20.3%)	16 (20.3%)	
Diabetes mellitus	20 (25.3%)	18 (22.8%)	0.853
Hyperlipidemia	30 (38.0%)	23 (29.1%)	0.312
**CLINICAL VARIABLES**			
SBP (mm Hg)	140.92 ± 20.41	147.03 ± 17.54	0.046*
DBP (mm Hg)	83.35 ± 10.99	82.29 ± 10.61	0.537
Glucose (mmol/l)	5.69 ± 2.28	5.34 ± 1.51	0.255
TC (mmol/l)	4.59 ± 1.27	4.30 ± 1.06	0.126
THcy (μmol/l)	13.80 ± 5.15	14.19 ± 6.81	0.686
Hs-CRP (mg/l)	10.31 ± 8.40	8.42 ± 6.45	0.115
**RADIOLOGIC DATA**			
No. of microbleeds	3.51 ± 9.65	13.05 ± 23.35	0.002*
No. of lacunes	0.81 ± 1.90	2.97 ± 3.00	<0.001**
Voxel count of DMVs (10^3^)	3.132 ± 0.820	4.220 ± 1.018	<0.001**

*WMH, white matter hyperintensity; MMSE, mini-mental state examination score; SBP, systolic blood pressure; DBP, diastolic blood pressure; TC, total cholesterol; THcy, total homocysteine; Hs-CRP, high-sensitivity C-reactive protein; DMVs, deep medullary veins*.

*0.001 < *P* < 0.05; **P < 0.001.

**Table 2:** **Multivariate logistic-regression analysis of risk factors for large corrected WMH volume (>25 ml)**.

	Univariate analysis			Multivariate analysis		
	OR	95% CI	*P* value	OR	95% CI	*P* value
Age (y)	1.013	0.988–1.038	0.310	1.011	0.981–1.042	0.467
No. of lacunes	1.501	1.255–1.796	<0.001**	1.348	1.114–1.631	0.002*
Voxel count of DMVs (10^3^)	3.725	2.344–5.920	<0.001**	3.229	1.986–5.251	<0.001**

*WMH, white matter hyperintensity; OR, odds ratio; DMVs, deep medullary veins*.

*0.001 < *P* < 0.05; **P < 0.001.

To understand the characteristics of patients with an increased visibility of the DMVs, we compared the baseline characteristics of WMH patients classified according to the number of voxels of DMVs dichotomized at the median (number of voxels >3.500 × 10^3^). As seen in Table 3, patients with an increased number of voxels of DMVs had a higher prevalence of lacunes and microbleeds and larger CWMHV.

**Table 3:** **Baseline characteristics of WMH patients classified according to the number of voxels of the deep medullary veins dichotomized at the median**.

Variable	Voxel count of DMVs		*P* value
	Low (≤3500, *n* = 79)	High (>3500, *n* = 79)	
Age (y)	66.11 ± 13.33	66.91 ± 12.24	0.696
Female	38 (48.1%)	40 (50.6%)	0.874
Years of education (y)	6.76 ± 4.59	7.76 ± 4.57	0.171
MMSE	26.71 ± 3.10	24.95 ± 4.01	0.135
**PAST MEDICAL HISTORY**			
Hypertension			0.632
No	21 (26.6%)	17 (21.5%)	
Yes, with medication	41 (51.9%)	47 (59.5%)	
Yes, without medication	17 (21.5%)	15 (19.0%)	
Diabetes mellitus	20 (25.3%)	18 (22.8%)	0.853
Hyperlipidemia	28 (35.4%)	25 (31.6%)	0.736
**CLINICAL VARIABLES**			
SBP (mm Hg)	145.25 ± 18.99	142.70 ± 19.47	0.405
DBP (mm Hg)	82.42 ± 11.48	83.23 ± 10.09	0.638
Glucose (mmol/l)	5.52 ± 2.14	5.52 ± 1.71	0.992
TC (mmol/l)	4.41 ± 1.19	4.49 ± 1.16	0.659
THcy (μmol/l)	14.08 ± 7.03	13.91 ± 4.85	0.861
Hs-CRP (mg/l)	10.44 ± 8.38	9.29 ± 7.43	0.173
**RADIOLOGIC DATA**			
No. of microbleeds	2.06 ± 3.95	14.49 ± 24.31	<0.001**
No. of lacunes	1.13 ± 2.03	2.66 ± 3.11	<0.001**
Corrected WMH volume (ml)	24.36 ± 23.81	46.28 ± 28.21	<0.001**
Corrected PVH volume (ml)	14.49 ± 13.84	23.84 ± 12.69	<0.001**
Corrected DWMH volume (ml)	9.87 ± 12.33	22.44 ± 20.87	0.002*
Regional WMH volume (ml)	5.97 ± 4.18	15.09 ± 9.90	<0.001**

*WMH, white matter hyperintensity; DMVs, deep medullary veins; MMSE, mini-mental state examination score; SBP, systolic blood pressure; DBP, diastolic blood pressure; TC, total cholesterol; THcy, total homocysteine; Hs-CRP, high-sensitivity C-reactive protein; PVH, periventricular hyperintensity; DWMH, deep white matter hyperintensity*.

*0.001 < *P* < 0.05; **P < 0.001.

### Relationship between the DMVs and WMHs in different regions

Significant correlations were both found between voxel number of DMVs and corrected PVH volume (Pearson *r* = 0.556, *P* < 0.001; partial correlation coefficient = 0.492, *P* < 0.001, adjusting for age, sex, years of education, and number of microbleeds and lacunes), and corrected DWMH volume (Pearson *r* = 0.513, *P* < 0.001; partial correlation coefficient = 0.431, *P* < 0.001). Interestingly, we found that voxel number of DMVs had much higher correlation with coregistered regional WMH volume (Pearson *r* = 0.710, *P* < 0.001; partial correlation coefficient = 0.670, *P* < 0.001; Figure 3B) than those with CWMHV of the whole brain.

## Discussion

In the current study, we found significantly increased visibility of the deep medullary venous vasculature in patients with WMHs as compared to controls. We also found an independent association between the visibility of DMVs and CWMHV, especially the WMH volume around the DMVs, suggesting that cerebral periventricular venous dilation or ischemia might be involved in the pathogenesis of WMHs.

Susceptibility-weighted imaging uses the application of filtered phase images to enhance contrast by means of susceptibility differences between tissues (Haacke et al., 2004). Due to the magnetic susceptibility difference between oxygenated and deoxygenated hemoglobin, venous vessels were visualized as hypointense on SWI venography. The visibility of the venous vessels on phase imaging thus depends on the de-oxygenation of the vessel (Rauscher et al., 2005). One might speculate that the increased visibility of the DMVs could be a result of increased cerebral metabolism. However, reduced cerebral metabolism has already been shown in subjects with large WMH volumes (Decarli et al., 1995). With quantitative perfusion MRI, reduced CBF has been demonstrated in white matter of WMH subjects (O’Sullivan et al., 2002). This reduced perfusion would lead to increased oxygen extraction from vessels experiencing reduced blood flow, thus resulting in greater visibility of venous structures by SWI. The presence of WMH may also facilitate a negative environment that increases the likelihood of DMVs through this channel. Patients with larger WMH usually have more lacunes, while lacunes most often result from arterial ischemia (Basile et al., 2006; Gouw et al., 2008), which may induce venous ischemia and lead to greater visibility of DMVs to some extent. Our finding of more lacunes among patients with increased visibility of the DMVs may support this assumption.

However, in the current study, the correlation of voxel count of DMVs and WMH volume was still significant after adjusting for the number of lacunes in either logistic-regression model or partial Pearson’s correlation analysis model. Thus, we hypothesized that increased number of voxels of DMVs might influence the severity of WMH. It is possible that the increased deep medullary venous vasculature on SWI in severe WMHs itself represents altered venous hemodynamics or venous vascular occlusions. A previous study of autopsy has found that periventricular venous collagenosis, which caused intramural thickening and stenosis, was strongly associated with WMHs (Moody et al., 1995). This venulopathy, through the dilatation of upstream venule beds (Schaller and Graf, 2004; Black et al., 2009), would be expected to contribute to the enhancement of susceptibility effects. Venous congestion with the development of enlarged, tortuous veins has been demonstrated to lead to partially reversible white matter T2 hyperintensity on MR images in patients with brain arteriovenous malformations after treatment (Willinsky et al., 1999; van den Berg et al., 2008). Recently, based on postmortem MRI and histopathologic methods, Black et al. proposed that venous collagenosis could dilate the veins and damage the myelin and axons with the consequent leakage of potentially toxic substances (Black et al., 2009). These were consistent with the pathological findings in regions of WMHs, including myelin pallor, tissue rarefaction, loss of myelin and axons, and mild gliosis (Erkinjuntti et al., 1996; Matsusue et al., 2006). Therefore, the development of WMHs can be promoted by the stenosis or occlusion of deep cerebral veins (Schaller and Graf, 2004). Moreover, there also exists the possibility that the iron content was increased within altered cerebral venous return (Singh and Zamboni, 2009), and this iron deposition might predispose to WMHs by activating the immune regulation and damaging the myelin in the white matter (Adams, 1988; Fornage et al., 2008). Venous outflow obstruction or retrograde venous hypertension may also lead to decreased CBF, venous ischemia, and hypoxia (Bederson et al., 1991). Therefore, our finding of the increased number of voxels of DMVs in WMHs may provide *in vivo* corroboration for the role of periventricular venular reflux in the pathogenesis of WMH.

In this study, both PVH and DWMH volume were found to be correlated with voxel count of DMVs, with relatively higher correlation coefficient with PVH. According to venous anatomy, supratentorial medullary veins are divided into DMVs and superficial medullary veins, which were not easy to measure by the current imaging technology (Huang and Wolf, 1964). Thus, the fact that part of DWMH was drained by the superficial medullary veins might explain the different correlations. Moreover, the coregistered regional WMH volume was found to have a much higher correlation with the number of voxels of DMVs than the CWMHV of the whole brain. We thus posit that DMVs may have a direct influence on the surrounding white matter. However, it needs to be proved by future follow-up studies.

Several studies have reported the association between WMHs and the number of microbleeds (Kato et al., 2002; Naka et al., 2004). We found that patients with increased number of voxels of DMVs had more microbleeds. However, the number of microbleeds did not independently influence the WMH volume. It is possible that microbleeds and WMHs may share the major risk factors or the common mechanisms, i.e., cerebral venous dilation, ischemia, and hypoxia. Further study to investigate the relationship between cerebral venous dilation and microbleeds may help to clarify it.

White matter hyperintensities are proved to be the major contributors to declined mentality and disability in the elderly. However, few have been shown to be specifically effective in the treatment of WMHs. Treatment trials for WMHs face major challenges, including relatively slow progression of the disease. Our current study provides the possibility of SWI as a radiological marker for the evaluation of the therapeutic effectiveness in WMHs. By investigating the number of voxels of DMVs, we may identify subjects at higher risk of developing severe WMHs. It may also be significant for the development of new drugs, such as those improving cerebral venous hemodynamics or increasing the venous tone, which may prevent the progression of WMHs.

There are limitations to our study. First, the signal to noise ratio (SNR) in SWI phase images is relatively lower, compared with those in mIP venograms. However, mIP images are lack of three-dimensional information of blood vessels. Moreover, its partial volume effect may reduce contrast and affect volumetric measurement due to the small size of DMVs. Second, segmentation and automated detection may misclassify venous structures and abnormal hypointense lesions. Although we manually removed the false venous areas, this step was relatively subjective. Future advanced algorithm of post-processing is needed to improve the detection of cerebral veins. Third, the step of coregistration had inherent artifact, especially for small structure (such as DMVs). We thus used continuous thin-slice (2 mm for both SWI and FLAIR) 3-T MRI scans and set the phase images as reference (unchanged) to minimize the effects of artifact. Fourth, our cohort only included Chinese patients at a single institution who had symptoms or distinct imaging changes, and the exclusion criteria was strict. Therefore, it may not represent the full spectrum of WMHs and limit the generalizability of our results. Fifth, we did not have long-term follow-up data for most patients to assess the natural progression of WMHs based on the DMVs. Prospective and longitudinal studies are needed to clarify if the venous insufficiency contributes to the progression of white matter damage and whether it is on the causal pathway between increased DMVs and increased risks of stroke and cognitive decline.

In summary, our findings of cerebral venous insufficiency in WMHs patients could help to elucidate the pathogenic mechanisms and progression of WMHs. The quantitative measures of detected number of venous structures on SWI may allow the clinician to closely monitor disease progression. Moreover, our results may open new avenues to explore cerebral venous therapies in WMHs, such as improving venous hemodynamics or increasing the venous tone.

## Author Contributions

Conceived and designed the experiments: Min Lou and Shenqiang Yan. Performed the experiments: Shenqiang Yan, Song Zhao, Jianzhong Sun, and Yuehan Lin. Analyzed the data: Shenqiang Yan, Jianzhong Sun, Xuting Zhang, Lusha Tong, and Min Lou. Contributed reagents/materials/analysis tools: Xuting Zhang, Jinping Wan, Chunhong Shen, and Min Lou. Wrote the paper: Shenqiang Yan and Min Lou.

## Conflict of Interest Statement

The authors declare that the research was conducted in the absence of any commercial or financial relationships that could be construed as a potential conflict of interest.
